# Electroacupuncture for psychogenic erectile dysfunction: A resting-state functional magnetic resonance imaging study exploring the alteration of fractional amplitude of low frequency fluctuation

**DOI:** 10.3389/fnhum.2023.1116202

**Published:** 2023-03-30

**Authors:** Yuqing Yang, Liu Qu, Linxuan Mu, Junpeng Yao, Chengguo Su, Qianhua Zheng, Huabin Zheng, Peihai Zhang, Ying Li

**Affiliations:** ^1^Acupuncture and Tuina School, Chengdu University of Traditional Chinese Medicine, Chengdu, Sichuan, China; ^2^Department of Acupuncture and Moxibustion, The Hospital of Chengdu University of Chinese Medicine, Chengdu, Sichuan, China; ^3^Department of Urology/Andrology, The Hospital of Chengdu University of Chinese Medicine, Chengdu, Sichuan, China

**Keywords:** resting-state fMRI, posterior cingulate cortex, default mode network, electroacupuncture treatment, psychogenic erectile dysfunction, fractional amplitude of low frequency fluctuation

## Abstract

**Background:**

Psychogenic erectile dysfunction (PED) can seriously affect emotional and marital wellbeing. Electroacupuncture (EA) seems an effective method for treating PED. However, the central mechanisms underlying PED and the beneficial effects of EA treatment are unclear. The purpose of this study was to explore the central mechanisms of PED and to examine the impact of EA on erectile function.

**Methods:**

We recruited 14 PED patients and 14 matched normal controls (NCs). PED patients underwent twice rs-fMRI scans, respectively, pre- and post-treatment. The NCs only completed one rs-fMRI scan. We used the fractional amplitude of low frequency fluctuation (fALFF) to compare spontaneous neural activity between the PED patients and NCs, and to examine the differences between the pre- and post-EA treatment scans in the PED patients.

**Results:**

Scores on the IIEF5, QEQ, and SEAR improved after EA treatment. Compared with the NCs, PED patients showed increased fALFF in the right posterior cingulate cortex (PCC), right dorsolateral prefrontal cortex (DLPFC), right supplementary motor area (SMA), and left middle occipital gyrus. Most of these regions are closely implicated in sexual inhibition. The results of the correlation analysis results indicated that the fALFF of the right PCC was negatively correlated with IIEF5 scores. After treatment, fALFF values were substantially lower in the left triangular part of the inferior frontal gyrus, right DLPFC, right SMA, bilateral PCC and the orbital part of the middle frontal gyrus, and higher in the left middle temporal gyrus and left caudate nucleus. These regions mainly belong to the default mode network (DMN), executive control network and primary sensory motor network. The results of the correlation analysis indicated a positive association between the changes in IIEF5 score and changes in the fALFF value in the right PCC after EA treatment.

**Conclusion:**

In conclusion, our study highlights that PED patients have abnormal patterns of activity in the right PCC, right DLPFC, and right SMA mainly involved in the DMN, executive central network, and sensory motor network which could lead to a higher levels of sexual inhibition. EA might regulate the process of sexual inhibition to improve erection function in PED patients probably by modulating spontaneous brain activity in the DMN, executive central network, and sensory motor network.

## Introduction

Erectile dysfunction (ED) refers to the failure of the male penis to maintain and (or) achieve sufficient erections to achieve a satisfactory sexual intercourse. ED is one of the most common forms of sexual dysfunction in men (McCabe et al., 2016). A cross-sectional study conducted in the United States showed that approximately 14% of sexually active men aged 18–31 years experienced mild to severe ED (Calzo et al., 2021). Another study suggested that the number of ED patients globally would reach 322 million by 2025 (Ayta et al., 1999). Although ED itself does not pose a direct threat to a patients’ life, it can seriously affect emotional and marital wellbeing, and thus endanger family harmony and social stability (Bacon et al., 2003; Ponholzer et al., 2010; Rinkûnienë et al., 2021). According to its pathogenesis, ED can be divided into psychogenic ED (PED), organic ED (secretory ED, venous ED, arterial ED, neural ED, etc.) and mixed ED. It is generally believed that PED is mainly caused by psychological or interpersonal factors such as anxiety, depression, lack of confidence, or problems in one’s intimate relationship(s), and is usually not accompanied by disease-related physical organic lesions (Chen et al., 2019; McMahon, 2019). PED has been found to account for 13–85.2% of ED patients under 40 years old (Zou et al., 2019; Pozzi et al., 2022).

Psychosexual therapy such as cognitive behavioral therapy and positive thinking are considered the preferred treatment methods for PED, but it often requires a high level of motivation and willingness to cooperate, the support and cooperation of the partner, and a longer treatment period (2–3 months on average) (Baum et al., 2000; Rew and Heidelbaugh, 2016). Oral phosphodiesterase type 5 inhibitors (PDE5i) are reliably safe and efficacious, and are usually used in PED clinical treatment (Muneer et al., 2014). However, some ED patients have to choose withdrawal because of a variety of adverse reactions and complications, such as headache, nausea, facial flushing, and muscle pain (Shamloul and Ghanem, 2013; Karakus and Burnett, 2020). Moreover, the long-term efficacy of PDE5i is poor (El-Galley et al., 2001; McMahon et al., 2006). Other treatment method include intracavernosal and intraurethral administration, vacuum erection devices, low-intensity extracorporeal shockwave therapy, and penile prosthesis implantation. These methods are usually aimed at organic ED rather than PED and have various side effects. For example, vacuum erection devices and low-intensity extracorporeal shock wave therapy may cause penile ecchymosis, pain, and discomfort. Intracavernosal administration may cause penile fibrosis, pain and priapism. These factors make them undesirable options for most PED patients (Shamloul and Ghanem, 2013; Rizk et al., 2018; Wang et al., 2022). Given the limitations of existing ED treatments, researchers are searching for new promising treatments (Li et al., 2017a).

Acupuncture, as an important method of traditional Chinese medicine, has become increasingly popular for the management of sexual dysfunction in men. This is because it has been found to have substantial curative effects with few adverse events (Abdi et al., 2021). A large number of clinical studies and meta-analyses have reported that acupuncture and electroacupuncture (EA) can significantly improve clinical symptoms in patients with mild to moderate ED, as well as enhance patient confidence, with no obvious side effects or safety concerns (Yaman et al., 1994; Engelhardt et al., 2003; Li et al., 2017a,b; Lai et al., 2019; Abdi et al., 2021). These studies indicated that acupuncture might ameliorate ED symptoms by regulating hypothalamic function and levels of reproductive hormones, as well as by stimulating local neuromuscular function (Wang et al., 2022). However, the potential neurobiological mechanisms by which acupuncture treatment could ameliorate the symptoms of PED are still unknown.

Several resting-state functional magnetic resonance imaging (rs-fMRI) studies have examined potential PED-related changes in brain structure and function. For instance, previous studies reported that PED patients had abnormal activity patterns in multiple areas such as the precentral gyrus, lateral cerebellum, insula, anterior cingulate gyrus, dorsolateral prefrontal cortex (DLPFC), and prefrontal lobe. These brain regions are associated with sensory integration, motor imagery and motor execution, and inhibition control (Wang et al., 2017; Jin et al., 2018; Yin et al., 2020a,b). Other studies reported that alterations in the default mode network (DMN), salience network, primary sensory motor network, and limbic system could be associated with the altered emotional and cognitive function observed in PED patients (Cera et al., 2014; Wang et al., 2017; Jin et al., 2018; Yin et al., 2020a). In sum, PED involves multi-level changes in brain activity in regions associated with cognitive and psychosocial functions such as attention, evaluation, emotion, and sensorimotor activity (Zhang J. et al., 2022).

Several studies have indicated that rs-fMRI is both an effective method for detecting the aberrant neural representations associated with PED and a useful way to evaluate the central integration mechanisms of acupuncture for PED treatment (Zhang et al., 2021a; Zhao et al., 2022). The fractional amplitude of low frequency fluctuation (fALFF) measures the ratio of the power spectrum at a low-frequency range to that of the whole frequency range. This is one of the most commonly used assessments of regional spontaneous brain activity during rs-fMRI, and has high sensitivity and specificity (Zou et al., 2008). Here, we observed the alterations in brain imaging of PED as well as the effects of EA on patients with PED, and analyzed the correlations between fALFF neuroimaging findings and PED-related clinical outcomes to explore the central mechanisms of PED and the central mechanisms characteristics of EA for PED.

## Materials and methods

### Participants

A total of 14 male patients with PED (mean age = 28.5 ± 4.54 years) and 14 male normal controls (NCs) (mean age = 27.2 ± 4.51 years) were recruited from 1 March 2021 to 30 October 2022. Both groups were managed by Chengdu university of Traditional Chinese Medicine. PED patients were from the outpatients department of andrology of Hospital of Chengdu university of Traditional Chinese Medicine and NCs group were volunteers recruited through advertisement. The inclusion criteria for PED were as follows: (a) meeting the Diagnostic and Statistical Manual of Mental Disorders-Fifth Edition (Segraves, 2010); (b) the scores of 5-item International Index of Erectile Function (IIEF5) between 8 and 21 (Rosen et al., 1997); (c) the normal night erection evaluated by RigiScan device (Liu et al., 2020); (d) duration of PED more than 3 months (Male Science Society of Chinese Medical Association, 2016). The inclusion criteria for NCs were IIEF-5 score >21, and had no organic or mental or nervous system disease. Moreover, all subjects must meet the following inclusion criteria: between 20 and 50 years old, right-handedness, a stable heterosexual relationship for at least 6 months, regular sexual life (once a week), and at least 9 years of education. Specially, all subjects underwent a complete medical history, physical examination, blood biochemistry.

Exclusion criteria of both groups were as follows: (a) taking PDE5i or other drugs that may affect the judgment of acupuncture efficacy in 4 weeks before enrollment and during treatment; (b) having other acute, advanced, severe, or unstable diseases such as heart disease, hypertension, cancer and so on; (c) having a history of serious psychosocial disorders (standard score of SAS ≥70 or standard score of SDS ≥70); (d) having severe cranial anatomic asymmetry or definite lesions; (e) contraindications for MRI examination; and (f) those who failed to cooperate in completing the corresponding evaluation and inspection.

### Intervention

**FIGURE 1:**
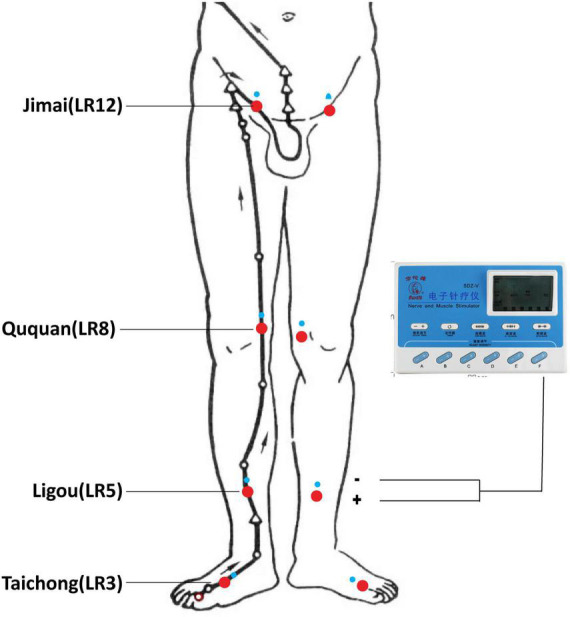
Locations of selected acupoints. The acupoints were Taichong (LR3), Ligou (LR5), Ququan (LR8), and Jimai (LR12).

Chinese medicine theory (TCM) hold that the normal function of liver meridian is very important for the initiation and maintenance of erection. Furthermore, the TCM pathogenesis of PED is mainly due to emotional dysfunction and liver qi stasis leading to impotence. Therefore, to better regulate the function of liver meridian, we selected four acupoints of liver meridian including Taichong (LR3), Ligou (LR5), Ququan (LR8), and Jimai (LR12). The above acupoints are selected on both sides (Figure 1). The detailed information for the locations and insertion methods of the acupoints is displayed in Supplementary Table 1. The PED patients take the supine position. Acupuncturists routinely disinfect their hands and the surround skin of chosen acupoints of patients and quickly insert needles into the acupoints. Then the needles should be twirled and lift in order to elicit “deqi” sensation. Then, an auxiliary needle (φ0.16 mm × 13 mm, needling depth about 5 mm) is inserted at the proximal end of meridian at each acupoint. EA procedures will be carried out with electronic instruments (Hwato SDZ-II, Suzhou Medical Supplies Factory Co., Ltd, China). The main acupoint is connected to the negative electrode, the auxiliary needle is connected to the positive electrode. The EA waveform is set to continuous wave, the frequency is 2 Hz, and the current intensity is 0.5–1.0 mA. Patients will receive a total number of 24 EA treatments, with the frequency of three times per week for 8 weeks. All acupuncture treatments are performed by qualified practitioners who have undergone rigorous clinical and operational training.

### Clinical outcome measurements

We used the IIEF5 to assess erection function of patients with PED, as our primary outcome measure. The IIEF5, a self-reported instrument for the assessment of male sexual function (Rosen et al., 1999), is advised as the important tool for screening and diagnostic ED (Cappelleri and Rosen, 2005) and has been linguistically validated in 32 languages (Rosen et al., 2002). IIEF5 has a score that ranges from 5 to 25, generally, and a score <22 was considered suggestive for an erectile dysfunction. Quality of erection questionnaire (QEQ) is a patient-reported measure, which specifically is used to evaluate men’s satisfaction with quality of erection from four sections: duration of erection, erection hardness, sensitivity or pleasure when stimulated, and overall erection quality and feeling (Porst et al., 2007). An increased QEQ score usually indicates an improvement in patient satisfaction with the quality of their erections. The SEAR is a 14-item questionnaire for measuring the sexual relationship, confidence, and self-esteem of ED (Althof et al., 2003). A increased SEAR score usually means that the patient’s self-confidence, self-esteem, and sexual relationships have improved. The self-rating anxiety scale (SAS) (Zung, 1971) and the self-rating depression scale (SDS) (Zung, 1965) are applied to measure the mental states, and higher scores of the two scales manifest the higher levels of anxiety and depression.

### Magnetic resonance imaging data acquisition

The participants received MRI examinations performed by the 3T magnetic resonance scanner (Discovery MR750, General Electric, Milwaukee, WI, United States) at the Medical Imaging Department, Hospital of Chengdu University of Traditional Chinese Medicine, with a 32 standard birdcage head coil. PED patients were collected MRI data twice totally, respectively, before EA intervention and within 1 week after EA treatment of 24 times. NCs were collected MRI data once without any intervention. The participants were instructed to keep their eyes closed and awake, and avoid psychological activity as much as possible during the scanning process. A head restraint was used to limit head movement, and earplugs were provided to reduce the noise. High-resolution three-dimensional T1-weighted MRI sequences used the following parameters: repetition time (TR) = 2700 ms, echo time (TE) = 3.39 ms, field of view (FOV) = 256 mm × 256 mm, flip angle = 7°, slice thickness = 1 mm. The parameters of a gradient-echo T2*-weighted echo planar imaging were as follows: scanning time = 8 min, resulting in 240 volumes, repetition time = 2000 ms, echo time = 30 ms, field of view = 240 mm × 240 mm, flip angle = 90°, 43 contiguous slices, with thickness = 4 mm, and matrix size = 64 × 64.

### Data pre-processing

Firstly, the organic DICOM data were converted into an analyzable NIFTI file format using the MRIcroN software programs.^1^ Then MRI data of the NIFTI format were preprocessed and analyzed by Statistical Parametric Mapping 12.0 (SPM12)^2^ and Data Processing Assistant for Resting-State fMRI (DPARSF) (Yan et al., 2016)^3^ working on MATLAB 2012a (Mathworks Inc. USA).

The preprocessing precodures were carried out with DPARSF, including (1) removed the first 10 time points; (2) temporal layer correction; (3) performed realignment; (4) spatial normalization (re-acquisition of 3 mm × 3 mm × 3 mm voxel images); (5) smoothed images with a 4 mm Gaussian kernel of full-width at half maximum; (6) removing linear trends; (7) regressed out nuisance covariates including white matter and cerebrospinal fluid signals, and head motion parameters [Friston 24 parameter model]; (8) performed temporal filtering (0.01–0.1 Hz); and (9) exclusion of participants whose head moved more than 3 mm on any axis or whose head rotated more than 3.

### Statistical analysis

#### Clinical data analysis

The SPSS software (version 26.0; IBM, Armonk, NY, United States) was applied for the statistical analysis of clinical data. Continuous, normally distributed variables were subjected to the two-sample *t*-test; continuous, non-normally distributed variables were analyzed using the Mann–Whitney *U*-test. The paired *t*-test was then applied to explore the changes in two groups of patients with PED before and after EA treatment. Differences were considered statistically significant at *P* < 0.05.

#### fALFF analysis

After image pre-processing, SPM12 and DPARSF were used for statistical analysis of mfALFF (original fALFF value/whole brain mean). First, the power spectrum was calculated by transforming the time series into the frequency domain. The square root of each frequency in the power spectrum was calculated, then, we obtained the mean square root across a low-frequency range (0.01–0.1 Hz), which was regarded as the ALFF. fALFF is the ratio of the sum of each frequency at the low-frequency range to that of the entire frequency range (Zou et al., 2008). Finally, the obtained spatial fALFF maps were normalized by dividing each voxel by the whole-brain fALFF mean to provide the mfALFF spatial map. Two-sample *t*-test was used for comparison between groups, and age and education year were included as covariates. Paired *t*-tests were used to quantitatively compare differences in mfALFF values between pre - and post-treatment using statistical parameter mapping. The false discovery rate (FDR) correction method was applied, and the significance of voxel levels was set at *P* < 0.05, minimum mass >10 voxels.

#### Correlation analysis

The Spearman correlation analysis was conducted to evaluate the relationships of fALFF values with clinical data in PED patients as well as the changes of fALFF values with the changes of clinical data. The significance level was set at two-tailed (*P* < 0.05).

## Results

### Demographics and clinical characteristics

There are 28 participants in our study, including 14 PED patients and 14 NCs with no significant differences between the two groups in terms of age, education, and BMI (*P* > 0.05). The demographic and clinical information are summarized in Table 1.

**TABLE 1:** Demographic and clinical conditions in patients with PED and NCs.

Characteristic	PED (*n* = 14)	NCs (*n* = 14)	t/Z	*P*
Age (years)	28.50 ± 4.54	27.20 ± 4.51	-0.752	0.459^b^
Education (years)	15.79 ± 2.08	15 ± 2	-1.018	0.318^b^
BMI	23.13 ± 2.39	22.99 ± 0.90	-0.220	0.828^b^
Duration (months)	14 (6.75, 24)^a^	/	/	/
IIEF5	15 (13, 16)^a^	24 (23, 24)^a^	-4.541	0.000^c^
SEAR	32.64 ± 8.29	73.37 ± 3.20	-8.154	0.000^b^
QEQ	45.67 ± 11.23	96.45 ± 2.79	-16.423	0.000^b^
SAS	41.34 ± 8.44	28.21 ± 2.34	5.607	0.000^b^
SDS	40.36 ± 5.91	30.71 ± 4.32	4.926	0.000^b^

PED, psychogenic erectile dysfunction; NC, normal controls; BMI, body mass index; IIEF5, 5-item international index of erectile function; SEAR, self-esteem and relationship; QEQ, quality of erection questionnaire; SAS, self-rating anxiety scale; SDS, self-rating depression scale. Except for the data “Duration” and “IIEF5,” other data were given as mean ± standard deviation.

^a^Median (interquartile range).

^b^*P*-values were calculated with two-sample *t*-test.

^c^*P*-values were calculated with Mann-Whitney *U*-test.

### Clinical characteristics of pre-and post-electroacupuncture

The clinical data of pre-and post-treatment are shown in Table 2. After EA treatment, the IIEF5, QEQ and SEAR scores significantly increased, with SAS scores decreasing in PED patients (*P* < 0.05).

**TABLE 2:** Clinical outcomes of pre- and post-electroacupuncture in PED group.

Characteristic	PED-pre	PED-post	t/Z	*P*
IIEF5	15 (13, 16)^a^	19 (16, 19.25)^a^	-3.640	0.000^c^
SEAR	32.64 ± 8.29	56.73 ± 13.25	2.127	0.043^b^
QEQ	45.67 ± 11.23	62.57 ± 11.71	-3.898	0.001^b^
SAS	41.34 ± 8.44	33.84 ± 11.71	2.369	0.026^b^
SDS	40.36 ± 5.91	35 ± 8.00	2.106	0.054^b^

IIEF5, 5-item international index of erectile function; SEAR, self-esteem and relationship questionnaire; QEQ, quality of erection questionnaire; SAS, self-rating anxiety scale; SDS, self-rating depression scale.

^a^Median (interquartile range).

^b^*P*-values were calculated with two-sample *t*-test.

^c^*P*-values were calculated with Mann-Whitney *U*-test.

### Resting-state functional magnetic resonance imaging data results

**FIGURE 2:**
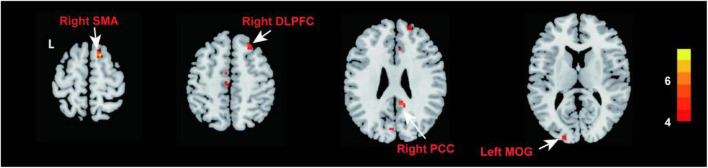
Comparison of fALFF between patients with PED and NCs (*P* < 0.05, FDR correction). Warm color represents an increase. fALFF, fractional amplitude of low frequency fluctuation; FDR, false discovery rate; PED, psychogenic erectile dysfunction; NCs, normal controls; PCC, posterior cingulate cortex; DLPFC, dorsolateral prefrontal cortex; MOG, middle occipital gyrus; SMA, supplementary motor area.

**FIGURE 3:**
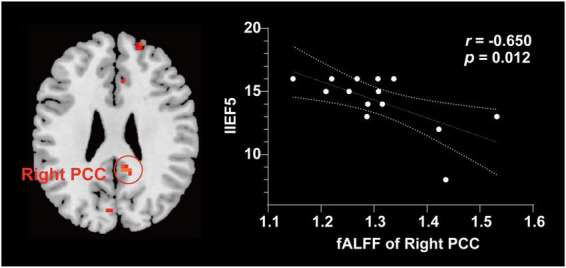
Correlation analysis between fALFF values and clinical characteristics in patients with PED. The increase of IIEF5 score was negatively correlated with the increase of fALFF value in right. fALFF, fractional amplitude of low frequency fluctuation; PCC, posterior cingulate cortex; IIEF5, 5-item international index of erectile function.

Compared with NCs, patients with PED mainly had increased mfALFF in the left middle occipital gyrus (MOG), the right posterior cingulate cortex (PCC), dorsolateral prefrontal cortex (DLPFC), and supplementary motor area (SMA) (Figure 2 and Table 3). In patients with PED, the IIEF5 score was negatively associated with the mfALFF values of the right PCC (*r* = −0.650, *P* = 0.012) (Figure 3).

**TABLE 3:** Brain regions with different fALFF values between PED patients and NCs.

	Brain regions	Brodmann area	Peak MNI coordinates			Cluster size	*T*-value
			x	y	z		
PED > NCs	Left MOG	17	–21	–96	12	10	4.649
	Right PCC	26	9	–45	27	15	4.932
	Right DLPFC	8	24	33	51	16	4.395
	Right SMA	6	15	15	63	16	6.510

Patients with pED manifested higher fALFF in right PCC, right MFC, right SMA, and left MOG than NCs (*P* < 0.05, FDR correction). fALFF, fractional amplitude of low frequency fluctuation; PED, psychogenic erectile dysfunction; NCs, normal controls; PCC, posterior cingulate cortex; DLPFC, dorsolateral prefrontal cortex; MOG, middle occipital gyrus; SMA, supplementary motor area; MNI, montreal neurological institute.

**FIGURE 4:**
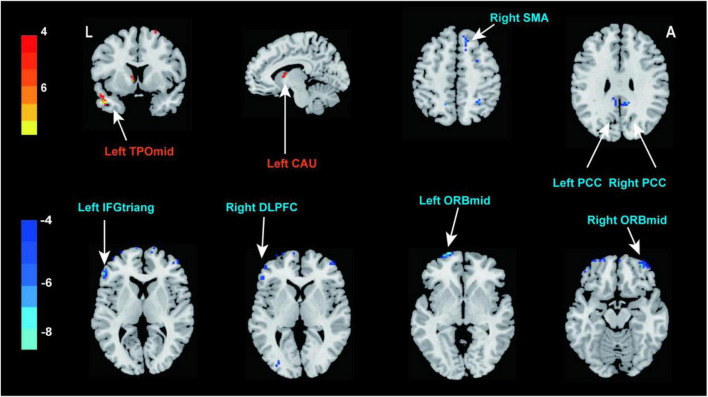
Brain responses to electroacupuncture treatment (*P* < 0.05, FDR correction). Warm color represents an increase and cool color represents a decrease, respectively. TPOmid, middle temporal gyrus; CAU, caudate nucleus; SMA, supplementary motor area; PCC, posterior cingulate cortex; IFGtriang, triangular part of inferior frontal gyrus; DLPFC, dorsolateral prefrontal cortex; ORBmid, orbital part of middle frontal gyrus.

**FIGURE 5:**
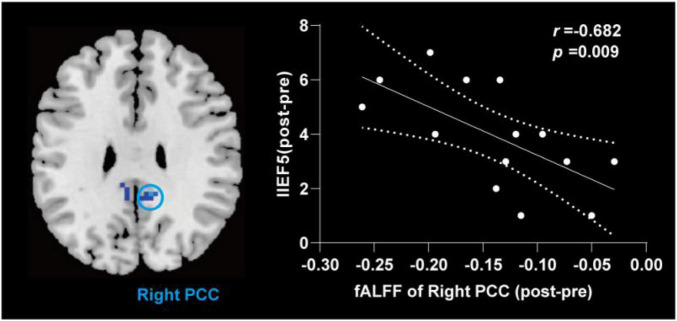
Correlation analysis between change value of fALFF and change of the IIEF5 score after electroacupuncture treatment in patients with PED. fALFF, fractional amplitude of low frequency fluctuation; IIEF5, 5-item international index of erectile function; PCC, posterior cingulate cortex; PED, psychogenic erectile dysfunction; NC, normal control; EA, after electroacupuncture treatment.

Compared with the pre-treatment, patients with PED after treatment mainly showed increased fALFF in left middle temporal (TPOmid) and caudate nucleus (CAU), as well as decreased fALFF in the triangular part of left inferior frontal gyrus (IFGtriang), the right DLPFC, SMA and bilateral PCC, the orbital part of middle frontal gyrus (ORBmid) (Figure 4 and Table 4). After EA treatment, the increase of IIEF5 score was positively correlated with the increase of fALFF value in the right PCC (Figure 5).

**TABLE 4:** Brain regions demonstrating altered fALFF values of PED patients after electroacupuncture.

	Brain regions	Brodmann area	Peak MNI coordinates			Cluster size	*T*-value
			x	y	z		
POST > PRE	Left TPOmid	20	–45	9	–30	27	7.734
	Left CAU	–	–6	9	0	13	6.406
POST < PRE	Left IFGtriang	45	–54	36	8	30	-7.836
	Right DLPFC	46	45	54	3	21	-5.561
	Right SMA	6	12	30	51	15	-5.794
	Right PCC	23	9	–42	30	13	-6.813
	Left PCC	23	–6	–48	27	18	-6.104
	Left ORBmid	11	–21	69	–3	25	-8.470
	Right ORBmid	–	33	54	–15	23	-7.288

After EA treatment, patients with PED manifested increased fALFF in left TPOmid and CAU, as well as decreased fALFF in left IFGtriang, right SMA and DLPFC and bilateral PCC, ORBmid (*P* < 0.05, FDR correction). POST, post-electroacupuncture; PRE, pre-electroacupuncture; TPOmid, middle temporal gyrus; CAU, caudate nucleus; SMA, supplementary motor area; PCC, posterior cingulate cortex; IFGtriang, triangular part of inferior frontal gyrus; DLPFC, dorsolateral prefrontal cortex; ORBmid, orbital part of middle frontal gyrus; MNI, montreal neurological institute.

## Discussion

This study newly investigated the potential central mechanism of the effect of EA in PED patients. Our study indicated four main findings. We found that (a) Compared with the NCs, patients with PED mainly exhibited fALFF in the right DLPFC, PCC, SMA, and left MOG. Remarkably, the fALFF values in the right PCC were negatively correlated with IIEF5 scores. (b) After EA treatment, erection function and anxiety state in PED patients was significantly ameliorated. (c) After EA treatment, PED patients showed decreased fALFF levels in the left IFGtriang, right DLPFC, SMA, bilateral PCC, and ORBmid, as well as increased fALFF levels in the left TPOmid and CAU. (d) In addition, the change in IIEF5 score after EA treatment was significantly and positively correlated with the change in fALFF value in the right PCC.

### Altered spontaneous brain activity occurred in PED patients

In this study, we found that PED patients exhibited abnormally increased activity in the right PCC. The PCC is thought to be a key node of the DMN that is a functional brain network and involves in many functions, such as self-referential introspection, self-esteem, comprehension, and remembering the past, among other cognitive activities (Buckner et al., 2008; Buckner and DiNicola, 2019; Wang et al., 2019). Previous studies have indicated that the DMN is an important brain network for regulating the male erection (Stoléru et al., 2012; Cheng et al., 2015; Yin et al., 2020a). Abnormal activity in certain regions of the DMN might lead to abnormal sexual arousal (SA) and behavior patterns, manifesting as PED (Cera et al., 2012; Zhao et al., 2015; Comninos et al., 2018) and other types of sexual dysfunction (Sumich et al., 2003; Poeppl et al., 2019). The PCC, as the posterior hub of the DMN (Wang et al., 2019), is involved in the storage and retrieval of emotional memories, as well as the integration of self-evaluation, perception, and attention (Leech and Sharp, 2014; Yin et al., 2020a). Several studies have reported that the PCC might be associated with the inhibition of sexual response (Cheng et al., 2015; Chen et al., 2017). For instance, the PCC was found to be activated during the inhibition of incoming sexual stimuli (cognitive sexual inhibition), which indicates that it might be implicated in a default, or tonic, sexual inhibitory mechanism (Rodriguez-Nieto et al., 2019). Moreover, Zhao et al. (2015) found that PED patients showed a reduction in cortical thickness in the PCC, which could contribute to the inhibition of SA. Cera et al. (2014) found that PED patients showed aberrant levels of connectivity in correspondence of the PCC/precuneus, which suggested disrupted processing of autobiographical and emotional memories in PED patients.

In the present study, PED patients exhibited increased fALFF values in the right DLPFC. The DLPFC is a core region of the executive central network (Seeley et al., 2007), primarily plays a fundamental role in cognitive control, decision-making, and attention (Cheng et al., 2015; Crone and Steinbeis, 2017). Previous studies have demonstrated that the DLPFC plays a critical role in male sexual inhibition and cognitive control (Spinella, 2007; Walter et al., 2007). For instance, Chen et al. (2017) found that PED patients showed altered path length and connectivity strength in the right DLPFC, and proposed that the disruptions in the topological structure of the DLPFC affected cognitive and emotional processes. Yin et al. (2020a) observed aberrant ALFF values in the left DLPFC, which indicates that patients with PED might have insufficient cognition and attention with respect to sexual targets, eventually leading to aberrant sexual inhibition. Another study also observed the aberrant activity patterns of DLPFC in patients with PED (Zhao et al., 2015). Actually, since the DLPFC is closely connected to some parts of the prefrontal cortex, it is considered a critical region for integrating sensory information with behavioral intentions, rules, and rewards (Cieslik et al., 2013). This functional integration maybe mediate conflicting decisions when responding to sexual stimuli (Cheng et al., 2015).

Moreover, we also found that the increased fALFF in the right SMA in PED patients. Generally, the SMA, which belongs to the primary sensory motor network, is thought to be involved in supporting sequence operations in various cognitive domains (Cona and Semenza, 2017). The activation of brain areas that mediate motor imagery, such as the SMA, could be involved in the cognitive processes associated with SA (Stoléru et al., 2012). In addition, studies reported the SMA might be involved in the negative motor network of the brain (Filevich et al., 2012; Rech et al., 2019). This motor network appears to be responsible for the inhibition of motor actions during their execution (Pinson et al., 2022), which might mediate the inhibition of the SA. Unterhorst et al. (2020) reported that activity in the SMA, prefrontal areas, dorsal anterior cingulate cortex, and other areas was associated with Sexual Inhibition Scales 1 scores, which indicates that sexual inhibition was caused by the threat of performance failure (i.e., performance anxiety).

The above finding indicates that PED patients might have abnormal inhibitory control of sexual responses. It is well known that there are brain mechanisms involved in sexual behavior. Previous studies have proposed a neurobehavioral model of male SA, which includes complicated components, such as perceptual, cognitive, motivational, emotional, inhibitory and autonomic components (Stoléru et al., 1999, 2003; Redouté et al., 2000, 2005; Georgiadis et al., 2012). Actually, the process of SA, generally involves the complex exchange and processing of information between and within brain networks such as the fronto-parietal, dorsal attention, default mode, salience, sensory motor, and visual networks (Chen et al., 2017). Poeppl et al. (2014) proposed that the deactivation of brain areas involved in introspective processes and social cognition releases intrinsic brain inhibition, which is necessary for SA. Accordingly, patients with PED appear to be affected by an over-inhibition of sexual response, which could make them attend more strongly to internal physical states rather than external erotic stimuli (Chen et al., 2019). This could eventually lead to higher levels of sexual inhibition and lower levels of sexual arousal and penile erection.

### Electroacupuncture modulates the brain region of PED patients

In this study, we found that EA could ameliorate IIEF5, QEQ, SEAR, and SAS scores in patients with PED, Thus, we obtained new evidence to support the idea that EA is an effective approach for treating PED. We applied the rsfMRI-fALFF technique to explore the potential central mechanism of the effect of EA for PED patients and found post-treatment changes in fALFF values in the brain region mainly were involved in the DMN (left TPOmid, left IFGtriang, right MFC, bilateral PCC, and bilateral ORBmid), executive central network (right DLPFC), and sensory motor network (right SMA). We also observed a general decrease in fALFF values. Moreover, the change in the fALFF values in the right PCC of PED patients was positively correlated with the change in the IIEF5 scores. Our studies manifests that the target brain regions of EA are mainly located in the DMN, executive central network, and sensory motor network. This result is consistent with a previously report that acupuncture is able to modulate the core brain regions of the DMN and autonomic nervous system which is dominated by limbic lobe structures (Dhond et al., 2008; Hui et al., 2009; Napadow et al., 2012). A review of neuroimaging studies about acupuncture also found that acupuncture regulated the abnormal functional activity of the DMN and sensorimotor network in areas of the brain associated with pain, emotion and memory.

As discussed earlier in this article, the perturbed brain activity in regions such as the PCC, DLPFC, and SMA might contribute to the over-inhibition of SA. This could be a crucial feature of abnormal central nervous activity in PED. We observed that the changed fALFF values in the right PCC, DLPFC, and SMA were reversed, from increasing to decreasing after EA. Our study has revealed that the right PCC, right DLPFC and right SMA are the overlapping brain regions, which means that they might be potential abnormal brain regions for PED and also be important targets for EA treatment. We speculate that EA improves the erection function in PED patients by modulating the aberrant activities of the PCC, SMA, and DLPFC to regulate self-introspective, social cognitive, and impaired psychosocial states. Zhang J. et al. (2022) have made similar findings that acupuncture could modulate function of DLPFC to improve cognitive symptoms of amnesic mild cognitive impairment. A rs-fMRI study of acupuncture on emotional disorders in patients with menstrual migraine without aura also found that acupuncture could regulate emotional disorders by modulating the frontal-limbic regions (Zhang et al., 2021b).

In addition, we found decreased post-treatment fALFF values in the bilateral ORBmid and left IFGtriang. These regions are parts of the DMN and are closely associated with SA. The orbitofrontal cortex (ORB) and IFG are thought to mediate the sexual inhibition, especially cognitive sexual inhibition, together with the DLPFC, PCC, and other brain regions (Cheng et al., 2015; Chen et al., 2017). Studies found that the abnormal patterns brain activity in the IFG of PED patients was likely to be related to dysfunction of sensorimotor function (Zhang X. et al., 2022). A previous study showed that the lesions in the ORB or frontal cortex often lead to impairments in socio-affective regulation including sexual inhibition (Baird et al., 2007; Cohen and Lieberman, 2010). In an rs-fMRI study of depression, the PCC had high functional connectivity with the lateral orbitofrontal cortex (Cheng et al., 2018). This connectivity was lower in medicated individuals (Rolls, 2019; Rolls et al., 2020). Altered fALFF values in the bilateral ORBmid, Left IFGtriang were only found after treatment, and we speculate that these changes may have arisen from abnormal activity of other brain regions.

Our results indicate that EA might regulate the process of sexual inhibition to improve erection function in PED patients probably by modulating spontaneous activity of certain brain regions in the DMN, executive central network, and sensory motor network.

### Limitations

This study had several limitations. First, we only explored the central mechanism of EA based on functional images, and in future studies, multimodal images could be used to comprehensively explore the central mechanisms. Second, we did not include a control group such as a waiting group or a sham acupuncture group. To further explore the efficacy of EA in PED patients, randomized controlled trials are needed. A larger sample size would also help to improve the stability and credibility of our results. Some objective indicators such as Rigiscan would also help to provide more evidence for the treatment of PED with acupuncture. Furthermore, we did not conduct follow-up rs-fMRI to examine the long-term effects of EA for treating PED. This is an important consideration for future research.

## Conclusion

In conclusion, our study newly explored the central mechanisms underlying the effects of acupuncture on PED symptoms. We found that excessive brain inhibition in PED patients was closely related to the activity of certain brain regions in the DMN, executive central network, and sensory motor network. EA appears to effectively modulate these abnormal targets, and correct for excessive inhibition, thus improving the IIEF5 score in PED patients. This conclusion needs to be further validated *via* large randomized controlled trials.

## Data availability statement

The raw data supporting the conclusions of this article will be made available by the authors, without undue reservation.

## Ethics statement

The studies involving human participants were reviewed and approved by the Sichuan Regional Ethics Review Committee on Traditional Chinese Medicine. The patients/participants provided their written informed consent to participate in this study. Written informed consent was obtained from the individual(s) for the publication of any potentially identifiable images or data included in this article.

## Author contributions

YL and PZ were responsible for the study concept and design. YY, LQ, and LM contributed to the acquisition of MRI data. JY, CS, and HZ assisted with data analysis and interpretation of findings. YY and QZ drafted the manuscript. All authors critically reviewed the content and approved the final version for publication.

## Footnotes

1
https://www.nitrc.org/projects/mricron

2
http://rfmri.org/DPARSF

3
http://www.fil.ion.ucl.ac.uk/spm
