# Supplementary material for: Electroacupuncture for psychogenic erectile dysfunction: A resting-state functional magnetic resonance imaging study exploring the alteration of fractional amplitude of low frequency fluctuation
Source: Front Hum Neurosci. 2023 Mar 30;17:1116202. doi: 10.3389/fnhum.2023.1116202 (PMC10098132; doi:10.3389/fnhum.2023.1116202)
Supplement: Supplementary file 1 [file Table_1.docx]

**SUPPLE Table 1 The locations and insertion details of the acupoints selected**

| Acupoint | Location | Specifications of needle | insertion depth |
| --- | --- | --- | --- |
| Taichong (LR3) | On the foot dorsum  between the first and second metatarsal bones. | φ0.35mm×25mm | depth of perpendicular insertion about 15-25mm |
| Ligou  (LR5) | In the medial side of the calf, 5.0 cun above the tip of the medial malleolus, the middle of the medial side of the tibia. | φ0.35mm×25mm | distance of transverse insertion upward along the Liver meridian about 15-20mm |
| Ququan (LR8) | In the medial knee, at the posterior edge of the medial femoral condyle, and in the depression of the anterior border of the insertions of semimembranosus muscle and semitendinosus muscle. When the knee is flexed, the point is at the medial end of the transverse popliteal crease. | φ0.30mm×40mm | depth of perpendicular insertion about 20-25mm |
| Jimai (LR12) | On the lateral side of the pubic tubercle, at the inguinal femoral artery beat, 2.5 cun beside the anterior median line. | φ0.30mm×25mm | depth of perpendicular insertion about 15-20mm |
